# Novel Application of Cultured Epithelial Autografts (CEA) with Expanded Mesh Skin Grafting Over an Artificial Dermis or Dermal Wound Bed Preparation

**DOI:** 10.3390/ijms19010057

**Published:** 2017-12-25

**Authors:** Sadanori Akita, Kenji Hayashida, Hiroshi Yoshimoto, Masaki Fujioka, Chikako Senju, Shin Morooka, Gozo Nishimura, Nobuhiko Mukae, Kazuo Kobayashi, Kuniaki Anraku, Ryuichi Murakami, Akiyoshi Hirano, Masao Oishi, Shintaro Ikenoya, Nobuyuki Amano, Hiroshi Nakagawa, Nagasaki University plastic surgeons group

**Affiliations:** 1 Department of Plastic Surgery, Wound Repair and Regeneration, School of Medicine, Fukuoka University, Fukuoka 814-0180, Japan; 2 Department of Plastic and Reconstructive Surgery, Nagasaki University Graduate School of Biomedical Sciences, Nagasaki 852-8523, Japan; tokimayu122710130311@gmail.com (K.H.); hy671117@nagasaki-u.ac.jp (H.Y.); csenju@nagasaki-u.ac.jp (C.S.); akiyoshi@nagasaki-u.ac.jp (A.H.); moishi999@hotmail.com (M.O.); 3 Division of Plastic and Reconstructive Surgery, Shimane University Hospital, Shimane 693-0021, Japan; 4 Department of Plastic and Reconstructive Surgery, National Hospital Organization Nagasaki Medical Center, Nagasaki 856-8562, Japan; mfujioka@nagasaki-mc.com (M.F.); moroshin.1760mm@hotmail.co.jp (S.M.); 5 Department of Plastic and Reconstructive Surgery, Fukuoka Tokushukai Hospital, Fukuoka 816-0864, Japan; irs.mggg@nifty.com; 6 Department of Plastic and Reconstructive Surgery, Kitakyushu General Hospital, Kitakyushu 802-8517, Japan; mojapon1220@icloud.com; 7 Department of Plastic and Reconstructive Surgery, Ehime Prefectural Central Hospital, Ehime 790-0024, Japan; kobak@silver.plala.or.jp (K.K.); hnakagawa@diary.ocn.ne.jp (H.N.); 8 Department of Plastic and Reconstructive Surgery, Sasebo City General Hospital, Sasebo 857-0056, Japan; anraku7@yahoo.co.jp; 9 Department of Plastic and Reconstructive Surgery, Yamaguchi Prefectural Grand Medical Center, Osaki 747-8511, Japan; rychi@ymghp.jp (R.M.); amanonobu1975@gmail.com (N.A.); 10 Department of Plastic and Reconstructive Surgery, Matsue Red Cross Hospital, Matsue 690-8506, Japan; ikesin09201225@yahoo.co.jp

**Keywords:** split-thickness skin grafting, cultured epithelial autografts (CEA), assessment of scar quality, generalized estimating equation (GEE), generalized linear mixed model (GLMM)

## Abstract

Cultured epithelial autografts (CEA) with highly expanded mesh skin grafts were used for extensive adult burns covering more than 30% of the total body surface area. A prospective study on eight patients assessed subjective and objective findings up to a 12-month follow-up. The results of wound healing for over 1:6 mesh plus CEA, gap 1:6 mesh plus CEA, and 1:3 mesh were compared at 3, 6, and 12 months using extensibility, viscoelasticity, color, and transepidermal water loss by a generalized estimating equation (GEE) or generalized linear mixed model (GLMM). No significant differences were observed among the paired treatments at any time point. At 6 and 12 months, over 1:6 mesh plus CEA achieved significantly better expert evaluation scores by the Vancouver and Manchester Scar Scales (*p* < 0.01). Extended skin grafting plus CEA minimizes donor resources and the quality of scars is equal or similar to that with conventional low extended mesh slit-thickness skin grafting such as 1:3 mesh. A longitudinal analysis of scars may further clarify the molecular changes of scar formation and pathogenesis.

## 1. Introduction

Scars and scar formation have been extensively investigated on a molecular basis; however, clinical scars are of many different characteristics and there are lots of variations. Additionally, it is very difficult for experimental models to mimic clinical features, because many animals do not exhibit hypertrophic scars or keloid in natural healing patterns. Thus, careful understanding of human scars and development of novel therapeutic modalities in humans are essential. Multiple and various treatments are required for extensive burns including burn resuscitation, cardiovascular, respiratory, and renal support, nutritional support, infection control, pain control, and surgical resurfacing and reconstruction. These procedures and interdisciplinary approaches improve survival rates [1,2]. Treatments using cultured epithelial autografts (CEA) were used clinically for the first time in the 1970s and early 1980s [3]. CEA are now more widely used in the treatment of extensive burn wounds in burn care specialist centers [4,5].

Commercially processed CEA developed in Japan (J-TEC Autologous Cultured Epidermis, JACE^®^, Japan Tissue Engineering CO., Ltd. (J-TEC), Gamagori, Aichi, Japan) were accepted by the health insurance reimbursement policy for the treatment of severe burns covering more than 30% of the total body surface area (TBSA) [6]. After 20 years of CEA development, JACE^®^ was produced and is a Green-type autologous cultured epidermis similar to EPICEL in the United States [7]. However, the manufacturing process for JACE^®^ differs from that for EPICEL. In a previous study, JACE^®^ was implanted with an artificial dermis in order to reconstruct the dermis and grafted with JACE^®^ on meshed 6:1 split thickness autografts [8]. Although the outcomes achieved were very good and acceptable, further detailed analysis of autograft site was not described.

A few quantitative studies have been conducted on clinical changes in burn scars and longitudinal burn scar quantification according to prospective quantified clinical characteristics of patient-matching, after burn hypertrophic scar (HSc), donor site scar (D) and normal skin (N) using these instruments and each investigator measured three sites (HSc, D, N) in 46 burn survivors 3, 6, and 12 months after burns [9].

Expanded skin grafting has recently been combined with CEA for extensive burns and is beneficial for wound coverage in multicenter surveillance; however, the quality of wounds and the rationale for using CEA with split-thickness skin grafting currently remain unknown [6]. 

Therefore, we assessed longitudinal burn scar qualification of expanded split-thickness skin grafting using clinical and objective methods up to a 12-month follow-up.

## 2. Results

There was no problem in donor site of CEA wound healing in all cases.

### 2.1. Vancouver and Manchester Scar Scales at 6 and 12 Months

#### 2.1.1. Vancouver Scar Scale (VSS)

At 6 and 12 months, the total value of the modified Vancouver Scar Scale (VSS) was significantly lower in over 1:6 mesh plus CEA (JACE^®^) than that in gap 1:6 mesh plus CEA (JACE^®^) or 1:3 mesh (6.6 ± 1.79, and 4.1 ± 1.35; 5.8 ± 1.41 and 5.6 ± 1.00 at 6 months; and 2.6 ± 1.04 and 5.1 ± 0.60 at 12 months, respectively, *p <* 0.01) (Table 1). 

The parameters of pigmentation, pliability, and vascularity in gap 1:6 mesh plus CEA, over 1:6 mesh plus CEA, and 1:3 mesh were 2.2 ± 0.70, 1.3 ± 0.50, 2.1 ± 0.50; 2.6 ± 0.70, 2.0 ± 0.60, 2.4 ± 0.60; 1.8 ± 0.50, 0.8 ± 0.40, 1.3 ± 0.43, at 6 months and 2.3 ± 0.30, 0.7 ± 0.40, 2.2 ± 0.40; 2.0 ± 0.70, 1.3 ± 0.40, 2.1 ± 0.50; 1.3 ± 0.40, 0.5 ± 0.30, 0.8 ± 0.30 at 12 months, respectively.

#### 2.1.2. Manchester Scar Scale (MSS)

At 6 and 12 months, the total value for the modified Manchester Scar Scale (MSS) was significantly lower in over 1:6 mesh plus CEA (JACE^®^) than in gap 1:6 mesh plus CEA (JACE^®^) or 1:3 mesh (12.7 ± 2.20 and 8.7 ± 1.20; 10.9 ± 1.80 and 10.7 ± 1.90 at 6 months; and 7.3 ± 1.20 and 10.2 ± 1.10 at 12 months respectively, *p <* 0.01) (Table 1).

The parameter of color, matte/shiny, contour, distortion or VAS (visual analogue scale) was 2.7 ± 0.30, 1.8 ± 0.30, 2.3 ± 0.38; 1.5 ± 0.30, 1.3 ± 0.10, 1.3 ± 0.20; 1.7 ± 0.40, 1.1 ± 0.20, 1.5 ± 0.30; 2.1 ± 0.40, 1.6 ± 0.30, 2.0 ± 0.36; 4.8 ± 1.00, 3.0 ± 0.60, 3.7 ± 0.78, gap 1:6 mesh plus CEA, over 1:6 mesh plus CEA, and 1:3 mesh, at 6 months, and 2.4 ± 0.30, 1.5 ± 0.30, 2.2 ± 0.20; 1.1 ± 0.10, 1.2 ± 0.10, 1.3 ± 0.20; 1.3 ± 0.30, 1.0 ± 0.00, 1.2 ± 0.20; 1.9 ± 0.30, 1.3 ± 0.20, 1.9 ± 0.20; 4.0 ± 1.10, 2.3 ± 0.60, 3.6 ± 0.60, gap 1:6 mesh plus CEA, over 1:6 mesh plus CEA, and 1:3 mesh, at 12 months, respectively.

### 2.2. Longitudinal Data from a Cutometer, Mexameter, Moisture Meter, and Color Meter (Table 2, Table 3 and Table 4)

#### 2.2.1. Cutometer

Maximal extensibility (R0) was analyzed by Model 4 and viscoelasticity (R7) by Model 2. No significant differences were observed in R0 or R7 among the over 1:6 mesh plus CEA, gap 1:6 mesh plus CEA, and 1:3 mesh. R0 increased with time, whereas R7 decreased from 3 to 12 months in all treatments.

#### 2.2.2. Mexameter

The melanin index was analyzed by Model 4 and the hemoglobin index by Model 2. No significant differences were observed in either index among the over 1:6 mesh plus CEA, gap 1:6 mesh plus CEA, and 1:3 mesh. The melanin index increased with time in all treatments. The hemoglobin index in the over 1:6 mesh plus CEA increased, while gap 1:6 mesh plus CEA and 1:3 mesh decreased from 3 to 12 months in all treatments.

#### 2.2.3. Moisture Meter

Transepidermal Water Loss (TEWL) was analyzed by Model 2. No significant differences were observed in TEWL among the over 1:6 mesh plus CEA, gap 1:6 mesh plus CEA, and 1:3 mesh. TEWL decreased from 3 to 12 months in all treatments.

#### 2.2.4. Color Meter

Clarity, yellow, and red were analyzed by Models 4, 2, and 2, respectively. Clarity and yellow increased from 3 to 12 months in all treatments. Red in the gap 1:6 mesh plus CEA increased, while red in the over 1:6 mesh plus CEA and 1:3 mesh decreased in a time-dependent manner.

## 3. Discussion

CEA for the wound coverage of extensive burns are useful for life-saving [2,3,4], and recent advances in bioengineered matrices have not only contributed to wound bed preparation, but also facilitated the expansion of coverage [5]. In Japan, the commercially available and reimbursed CEA was reported and followed Green-type CEA [7], and their indications and usefulness were discussed [6]. When a burn wound bed is prepared with an artificial dermis or with dermis remained wound beds, highly expanded (1:6 ratio) skin grafting with CEA may achieve similar healing to that with usual ratio skin grafting, such as a 1:3 mesh [8]. Too expanded mesh skin grafting such 1:6 may result in the irregularity or discoloration due to lack of skin component and thus scar formation, especially in the gap. However, the quality of wound healing, scars, and quality of life of patients, including the range of motion, texture, color, or function of reconstruction sites, have not yet been investigated. In the present study, the criteria, particularly of total burn surface areas, strictly followed the reimbursement category of TBSA of 30% or more, resulting in fewer cases being enrolled and precise each patient’s body surface is calculated and the surface area used with CEA was also achieved. In Japan, due to significant decreases in heavy industry production or maybe effective safety systems in industry or the environment, the number of patients with extensive burns has decreased. More than 90% of burn victims have total burn surface areas of less than 30% (the registry system of the Japanese Society for Burn Injuries), and, thus, this multi-center study only examined eight patients over a three-year period. Although one patient died due to hepatic cancer after eight months, the remaining seven patients were analyzed for scar and wound healing qualities using objective analyses with a cutometer, mexameter, moisture meter, and color meter in addition to expert evaluations with VSS and MSS at 3, 6, and 12 months. Except severe scars, the cutometer, mexameter and color reliability were acceptable. Concurrent validity correlations with the modified VSS (mVSS) were significant except for the comparison of the mVSS pliability subscale and the cutometer maximum deformation measure comparison in severe scar [10]. Longitudinal wound evaluation by a mixed model regression analysis demonstrated redder at 3 and 6 months but normalized by 12 months [9]. We adopted GEE and GLMM in objective data analyses. When data for each subject was considered to be correlated, and correlated structure is incorporated into GEE analysis, variation for each subject can be analyzed by a linear mixed model, and this is generalized as GLMM [11,12,13].

All data showed no significant differences when pairing among the over 1:6 mesh plus CEA, gap 1:6 mesh plus CEA or 1:3 mesh at all time points. This may explain why an increased ratio of 1:6 mesh plus CEA can bring about equal or similar results to conventional 1:3 meshes. With extensive burns, the donor site is limited and, thus, a smaller donor surface with greater expansion may reduce morbidity and be more economically resourceful [14]. Expert evaluations at 6 and 12 months demonstrated significantly lower VSS and MSS, suggesting some interactions between expanded skin grafts and CEA that improved burn scar qualities.

A prospective randomized multicenter intra-patient comparative study showed significantly increased epithelization, patient and observer scores, and some objective analysis by Wilcoxon’s non-parametric analysis at 3 and 12 months [15].

The usefulness of combining highly expanded mesh split skin grafting with CEA may be supported by studies involving larger subject numbers and appropriate statistical analyses. Furthermore, inter-racial and cultural backgrounds may strongly influence the outcomes achieved. These detailed clinical findings may clarify the longitudinal molecular pathogenesis and healing mechanisms.

## 4. Materials and Methods

Between 10 September 2012 and 31 March 2015, a prospective multicenter clinical study on longitudinal extensive burn scar assessments was approved by the Internal Review Board (#12090390, 10 September 2012) in full accordance with the Declaration of Helsinki. After a verbal explanation to each patient and their family, written informed consent was obtained at enrollment to this study. Patients with severe extensive burns greater than 30% of the total burn surface area of second degree or third degree burns, age older than 20 years, and survival at least 6 months post-operatively were included. Ten patients were initially recruited; however, due to limitations and decreases in the number of severe burn patients, 8 were ultimately enrolled and analyzed.

Patients were aged between 46 and 70 years, with a mean of 52.4 years, and comprised 7 males and one female. The total burn surface area was between 40% and 50%, with a mean of 44.9 ± 4.49%, the prognostic burn index was between 56 and 114, with a mean of 88.1 ± 17.02. Two patients did not receive dermal template temporal coverage until CEA, JACE^®^ was developed, 5 were treated with Integra^®^ (Century Medical, Tokyo, Japan), and one was treated with Terudermis (Olympus Terumo Biomaterials, Tokyo, Japan) (Table 5 and Table 6). The CEA sized 80 cm^2^ and each patient body surface was calculated by the Japanese body surface equation [16]. The CEA was applied 4.8 to 12.1% of patient’s body surface (Table 6).

One patient died 8 months post-operatively due to hepatic cancer, while the remaining 7 patients were followed up until 12 months.

**Figure 1:**
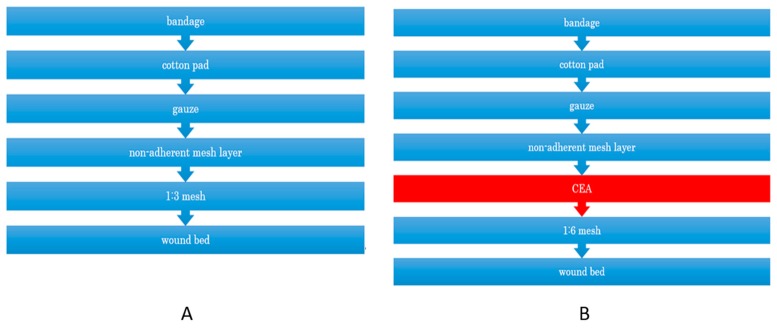
Schematic cross-section of 1:3 mesh without CEA (JACE^®^), (**A**) and 1:6 mesh plus CEA (JACE^®^) coverage, (**B**).

Intermediate split-thickness skin autografts, 15/1000-inch, were harvested with an electric dermatome and mesher (Zimmer Biomet, Dover, OH, USA) and expanded to a ratio of 1:3 or 1:6. The 1:3 mesh is frequently used clinically and heals in approximately 4 days, whereas the 1:6 mesh takes more than 17 days to heal [13], which is covered by CEA (JACE^®^) to simulate wound healing by factors from CEA or through an interaction between CEA and mesh skin grafting (Figure 1). It takes 3 to 4 weeks before CEA is ready for clinical application and harvesting of the “donor” is 3–4 cm in length and 1–2 cm in width in full thickness skin distant from the burns.

Patients underwent assessments 6 and 12 months after healing using VSS, which evaluates height, pliability, vascularity, and pigmentation on a scale of 0 to 13 [17], and MSS [18] and VSS were evaluated in a blind manner among 12 Nagasaki University plastic surgeons, who are all board certified, and independently by photographs. In VSS, “height” and “texture” in MSS were deleted because they cannot be assessed in a 2-dimensional analysis.

The following parameters were repeatedly and longitudinally evaluated in patients 3, 6, and 12 months after healing.

### 4.1. Moisture Meter

A compact and portable moisture meter was used (ASA-M2; Asahi Biomed, Yokohama, Japan). It only weighed 250 g (200 g for the power supply and 50 g for the hand piece) and had the ability to detect TEWL using an effective contact coefficient [19], water quantity, and the thickness of the stratum corneum skin layer by formulating the value of the effective contact coefficient, evaluated by electrolytes in the stratum corneum layer. The principle of this machine is to record and analyze conduction susceptibility using a low-frequency (160 Hz) alternate currency and to detect conduction admittance using a high frequency (143 kHz) alternate current.

The proposed formula is as follows:

Skin conductance (μc) = Effective contact coefficient (%) × Quantity of water (μS).

Low and high frequency electric voltages were added to effectively enable these formula factors. The round probe of the hand piece was 5 mm in diameter and detection was set to 5 s after probe contact with the participant in order to stabilize the electrodes and skin condition. The measurement of each contact point was always perpendicular to the participant, thereby avoiding unnecessary pressure or loading; it was repeated 5 times. The mean value of 3 adjacent points at least 10 mm apart and 20 mm from the edge of intact skin was assessed following the manufacturer’s instructions.

Measurements were performed by 2 of the authors who are very familiar with this system. All data were immediately transferred to a personal computer for further analyses.

In written informed consent, there was a description of data collection for all patients, and no complications occurred as a result of moisture meter measurements.

### 4.2. Color Meter

A color meter was used to assess scar clarity (L): “a” is red in color when the value is positive and green when it is negative, and “b” is yellow in color when the value is positive and blue when it is negative. The hand-held color meter weighed 420 g, including batteries, for the main body of the system and 110 g for the hand-piece probe and color analyzer (NF-333; Nippon Denshoku, Osaka, Japan). The light source was a multicolored LED. All data were easily transferred to Microsoft Excel 2013 files on a laptop computer via a data connector, and the differentials of each polarized color criterion parameter (L, a, and b) were standardized with the surrounding intact skin. The delta ratio of each parameter was then compared and statistically analyzed. The measurement of each point was always perpendicular to the scar and was repeated 5 times immediately after touching the scar surface. The mean value of 3 adjacent points at least 8 mm apart and 12 mm from the edge of intact skin was assessed in a single room. The accuracy of this system is traceable to the standard of the National Institute of Standards and Technology, Gaithersburg, MD. The accuracy of this function is based on the choice of optical filters, which are assessed by an optimization criterion using a combination of methodologies from differential geometry with a statistical error analysis. The magnitude of errors associated with the optimal filters was previously reported to be typically half that for typical RGB filters in a 3-parameter model of human skin coloration [20]. A relatively simple and easy-to-use skin chromatometer was employed to assess temporal changes after skin grafting, with multiple relevant factors such as age, the type of skin grafting, anatomical differences at the donor site or recipient site, and the Fitzpatrick skin type [21].

### 4.3. Cutometer

The cutometer MPA 580 (Courage + Khazaka Electronic GmbH, Cologne, Germany) was used to evaluate skin elasticity parameters 1 year after complete wound healing. The cutometer has the ability to measure the vertical deformation of skin by suctioning into a round probe that is 6 mm in diameter. A vacuum load of 500 mbar was used over the skin (or scar) surface for 1 s, followed by normal pressure for 1 s. Each measurement was repeated 3 times, and the mean value of 4 adjacent points at least 6 mm apart and 12 mm from intact skin was assessed. As discussed and reported previously, 2 parameters of the cutometer were used in the present study. Uf (depicted as R0) represents the maximal skin (or scar) extension of the deformation at the end of the vacuum period. Ur/Uf (R7) represents the ratio of retraction (Ur) to maximal extension (Uf) and reflects the elasticity of the measured skin [22,23]. These measurements were performed by 3 authors at 25 °C and 50% humidity with air conditioning to standardize patient conditions at the completion of wound healing and after 3 and 6 months.

### 4.4. Mexameter

The mexameter (MX18, Courage & Khazaka Electronic GmbH, Köln, Germany) quantifies scar erythema and melanin based on the tissue’s narrow wavelength light absorption. The probe has 16 light-emitting diodes that send 3 defined wavelengths of light (568, 660, and 880 nm). A receiver then measures the light reflected by the skin. Since the quantity of emitted light is known, the absorption rate of defined wavelengths may be ascertained, which are selectively absorbed by melanin (660 nm) pigments or hemoglobin (568 nm). The measurement area is 5 mm in diameter, although the total surface contacted by the probe is 2 cm in diameter. Similar to the cutometer, the central portion of the mexameter is spring-mounted to maintain constant pressure on the skin. In each measurement, the probe was held perpendicular to the skin. It lightly touched the skin surface, without the outer ring making contact, activating the light emitter. The reflected light was measured by the receiver and the erythema and melanin index (range 1–1000) was immediately displayed on the console; therefore, the probe only remains in contact with the skin for several seconds [10].

**Figure 2:**
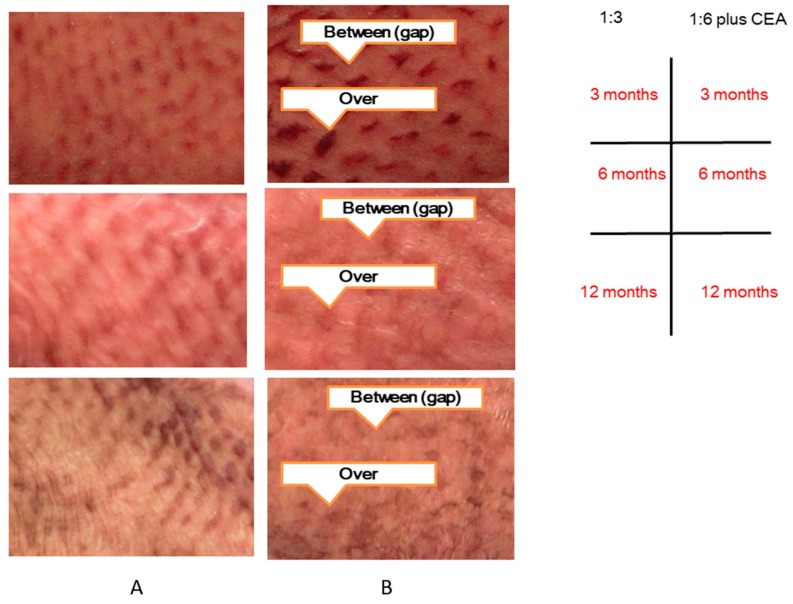
Measurement points of the gap 1:6 gap mesh plus CEA or over 1:6 mesh plus CEA (**A**) and 1:3 mesh (**B**).

Evaluator indices of VSS and MSS as well as objective measurements from the moisture meter, color meter, cutometer, and mexameter were obtained in the gap 1:6 mesh plus CEA (JACE^®^), over 1:6 mesh plus CEA (JACE^®^), and 1:3 mesh (Figure 2).

### 4.5. Statistical Analysis

Regarding VSS and MSS, results are expressed as the mean ± Standard errors (SE). Data among groups were evaluated using an independent *t-*test. All tests were 2-tailed, and the significance of differences was defined as *p* < 0.05. All analyses were performed using IBM SPSS, Statistics, and version 21 (Japan IBM, Tokyo, Japan).

Since repetitive and longitudinal data were obtained, GEE or GLMM was used in statistical analyses for the moisture meter, color meter, cutometer, and mexameter. When data for each subject was considered to be correlated, and correlated structure is incorporated into GEE analysis, variation for each subject can be analyzed by a linear mixed model, and this is generalized as GLMM.

There were 4 models (Table 2) and data from each time point were expressed as the mean ± SE (Table 3). Data analyses were performed with SAS version 9.2 (SAS Institute Inc., Cary, NC, USA) (Table 4).

## 5. Conclusions

When a burn wound bed is prepared with an artificial dermis or with dermis remained wound beds, highly expanded (1:6 ratio) skin grafting with CEA may achieve similar healing to that with usual ratio skin grafting, such as a 1:3 mesh. At 6 and 12 months, over 1:6 mesh plus CEA achieved significantly better expert evaluation scores by the Vancouver and Manchester Scar Scales (*p <* 0.01). Extended skin grafting plus CEA minimizes donor resources and the quality of scars is equal or similar to that with conventional low extended mesh slit-thickness skin grafting such as 1:3 mesh.

## Figures and Tables

**Table 1:** The Vancouver Scar Scale (VSS) and the Manchester Scar Scale (MSS), at 6 and 12 months among gap 1:6 mesh plus CEA, over 1:6 mesh plus cultured epithelial autografts (CEA), and 1:3 mesh.

Graft Type	6 Months		12 Months	
	VSS (Mean ± SE)	MSS (Mean ± SE)	VSS (Mean ± SE)	MSS (Mean ± SE)
1:6 gap + CEA	6.6 (0.60)	12.7 (0.81)	5.6 (041)	10.7 (0.70)
1:6 over + CEA	4.1 (0.50) **	8.7 (0.40) **	2.6 (0.40) **	7.3 (0.41) **
1:3 skin grafting	5.8 (0.50)	10.9 (0.61)	5.1 (0.20)	10.2 (0.40)

** *p* < 0.01.

**Table 2:** Models for generalized estimating equation (GEE) and generalized linear mixed model (GLMM) analyses.

	Linearity	Intercept	Slope	Selected Statistics
Model 1	non-linear	fixed	fixed	GEE
Model 2	linear	fixed	fixed	GEE
Model 3	linear	random	random	GLMM
Model 4	linear	random	fixed	GLMM

**Table 3:** Value of each parameter (3, 6 and 12 months).

Parameter	Over 1:6 Mesh Plus CEA (Mean ± SE)	Gap 1:6 Mesh Plus CEA (Mean ± SE)	1:3 Mesh (Mean ± SE)
**3 Months**			
Maximal extensibility (R0)	67.0 (±13.52)	62.6 (±8.48)	69.7 (±5.58)
Viscoelasticity (R7)	126.6 (±18.67)	93.7 (±13.06)	108.2 (±14.87)
Melanin Index	97.9 (±12.24)	118.1 (±15.80)	123.4 (±35.54)
Hemoglobin Index	133.2 (±11.89)	155.2 (±13.68)	144.7 (±14.16)
Transepidermal Water Loss (TEWL)	177.7 (±49.34)	160.3 (±32.44)	191.9 (±50.31)
Clarity	84.9 (±4.01)	79.9 (±3.21)	85.9 (±3.23)
Red	172.2 (±21.65)	181.7 (±19.87)	179.7 (±25.21)
Yellow	72.7 (±4.30)	66.9 (±3.99)	75.9 (±5.60)
**6 Months**			
Maximal extensibility (R0)	70.1 (±11.06)	67.5 (±9.48)	75.3 (±6.00)
Viscoelasticity (R7)	118.0 (±16.20)	87.3 (±11.23)	95.1 (±12.53)
Melanin Index	107.1 (±16.25)	121.2 (±17.18)	124.8 (±30.55)
Hemoglobin Index	135.2 (±12.38)	150.2 (±13.73)	141.0 (±13.83)
Transepidermal Water Loss (TEWL)	158.4 (±37.52)	155.1 (±20.30)	170.5 (±40.77)
Clarity	86.8 (±3.74)	82.7 (±3.15)	87.8 (±2.82)
Red	161.6 (±20.63)	182.8 (±17.61)	166.6 (±24.01)
Yellow	77.2 (±4.98)	70.6 (±3.73)	80.4 (±5.22)
**12 Months**			
Maximal extensibility (R0)	76.2 (±10.66)	77.5 (±15.15)	86.4 (±8.11)
Viscoelasticity (R7)	100.7 (±15.78)	74.4 (±9.54)	68.7 (±9.08)
Melanin Index	125.6 (±26.41)	127.4 (±21.68)	127.7 (±24.02)
Hemoglobin Index	139.1 (±15.74)	140.1 (±20.33)	133.6 (±15.99)
Transepidermal Water Loss (TEWL)	120.0 (±18.52)	144.7 (±33.15)	127.7 (±25.01)
Clarity	90.6 (±3.58)	88.2 (±3.35)	91.8 (±2.38)
Red	140.3 (±20.85)	185.1 (±22.83)	140.3 (±23.32)
Yellow	86.3 (±6.61)	77.9 (±4.16)	89.2 (±5.90)

**Table 4:** Model selection and statistical analysis.

	Selected Model	Comparision	Statistics among Groups
Maximal extensibility (R0) by a cutometer	Model 4	Over 1:6 vs. Gap 1:6	n.s. *p =* 0.7446
		Over 1:6 vs. 1:3	n.s. *p =* 0.9919
		Gap 1:6 vs. 1:3	n.s. *p =* 0.5604
Viscoelasticity (R7) by a cutometer	Model 2	Over 1:6 vs. Gap 1:6	n.s. *p =* 0.2059
		Over 1:6 vs. 1:3	n.s. *p =* 0.6296
		Gap 1:6 vs. 1:3	n.s. *p =* 0.3683
Melanin index by a mexameter	Model 4	Over 1:6 vs. Gap 1:6	n.s. *p =* 0.1653
		Over 1:6 vs. 1:3	n.s. *p =* 0.4409
		Gap 1:6 vs. 1:3	n.s. *p =* 0.8756
Hemoglobin index by a mexameter	Model 2	Over 1:6 vs. Gap 1:6	n.s. *p =* 0.1670
		Over 1:6 vs. 1:3	n.s. *p =* 0.3930
		Gap 1:6 vs. 1:3	n.s. *p =* 0.6004
Transepidermal Water Loss (TEWL) by a moisture meter	Model 2	Over 1:6 vs. Gap 1:6	n.s. *p =* 0.6926
		Over 1:6 vs. 1:3	n.s. *p =* 0.8510
		Gap 1:6 vs. 1:3	n.s. *p =* 0.5432
Clarity by a color meter	Model 4	Over 1:6 vs. Gap 1:6	n.s. *p =* 0.3013
		Over 1:6 vs. 1:3	n.s. *p =* 0.8910
		Gap 1:6 vs. 1:3	n.s. *p =* 0.1992
Red by a color meter	Model 2	Over 1:6 vs. Gap 1:6	n.s. *p =* 0.9463
		Over 1:6 vs. 1:3	n.s. *p =* 0.7801
		Gap 1:6 vs. 1:3	n.s. *p =* 0.7376
Yellow by color meter	Model 2	Over 1:6 vs. Gap 1:6	n.s. *p =* 0.4162
		Over 1:6 vs. 1:3	n.s. *p =* 0.6559
		Gap 1:6 vs. 1:3	n.s. *p =* 0.3040

Over 1:6, over 1:6 mesh plus CEA; gap 1:6, gap 1:6 mesh plus CEA, 1:3, 1:3 mesh; n.s.: not significant.

**Table 5:** Criteria for patients to participate in the present study. TBSA: total body surface area.

Inclusion Criteria
20 years of age or older
Acute full-thickness burn wounds that require widely meshed skin grafting
Minimal TBSA of 30% with full thickness wounds
Minimal study wound area of 100 cm^2^
Maximal study wound area of 300 cm^2^
Informed consent
Exclusion criteria
Immunocompromised patients or immunosuppressed physical conditions
Non-compliance by the patient, judged by medical experts
Active infected wounds
Known drug allergy

**Table 6:** Patient demographics. PBI: prognostic burn index. wks: weeks.

Case	Age	Sex	Burn Depth and Percent	TBSA	PBI	How to Reconstruct “Dermis“	Body Surface Area (m^2^)	Frequency of Grafts	Number of Sheets	Total CEA Covered BSA (%)	Percent Epithelialization at Four Weeks (%)	Prognosis
1	56	M	DDB 5% DB 40%	45	98	Terudermis	1.441	2	20 + 20	22.2	100	survive
2	59	M	DDB 30% DB 10%	40	84	none	1.6705	1	7	3.4	100	survive
3	31	M	DDB 30% DB 10%	40	56	none	1.8623	1	20	8.6	100	survive
4	41	M	DDB 10% DB 40%	50	86	Integra^®^	1.9984	1	32 + 12	17.1	50	survive
5	63	M	DDB 20% DB 25%	45	98	Integra^®^	1.5958	2	20 + 20	20.1	100	survive
6	53	F	DDB 35% DB 5%	40	78	Integra^®^	1.42	1	22	12.4	95	survive
7	70	M	DDB 10% DB 39%	49	114	Integra^®^	1.7985	2	24 + 11	15.6	50	deseased at 8 months
8	46	M	DDB 5% DB 45%	50	91	Integra^®^	1.98	2	30 + 30	24.2	95	survive

## Abbreviations

CEA	Cultured Epithelial Autografts
GEE	generalized estimating equation
GLMM	generalized linear mixed model
JACE^®^	Commercially processed CEA (J-TEC Autologous Cultured Epidermis, JACE^®^, Japan Tissue Engineering CO., Ltd. (J-TEC)
